# Evaluation of several serum interleukins as markers for treatment effectiveness in naïve HIV infected patients: A pilot study

**DOI:** 10.1371/journal.pone.0260007

**Published:** 2021-11-16

**Authors:** Cristian Jianu, Corina Itu-Mureşan, Cristina Drugan, Irina Filipescu, Adriana Violeta Topan, Mihaela Elena Jianu, Ioana Iulia Morar, Sorana D. Bolboacă

**Affiliations:** 1 Department of Medical Informatics and Biostatistics, Iuliu Hațieganu University of Medicine and Pharmacy, Cluj-Napoca, Romania; 2 Department of Immunosuppressed, Clinical Hospital of Infectious Diseases, Cluj-Napoca, Romania; 3 Department of Biochemistry, Iuliu Hațieganu University of Medicine and Pharmacy, Cluj-Napoca, Romania; 4 Department of Infectious Diseases, Iuliu Hațieganu University of Medicine and Pharmacy, Cluj-Napoca, Romania; 5 Department of Histology, Iuliu Hațieganu University of Medicine and Pharmacy, Cluj-Napoca, Romania; 6 Department of Pathophysiology, Iuliu Hațieganu University of Medicine and Pharmacy, Cluj-Napoca, Romania; University of Pittsburgh, UNITED STATES

## Abstract

In this observational pilot study, we investigated the impact of Dolutegravir, Raltegravir, Elvitegravir (Integrase Strand Transfer Inhibitors, INSTIs), or boosted Darunavir (a Protease Inhibitor, PI) in combination with two nucleoside reverstranscriptase inhibitors (Emtricitabine/Tenofovir disoproxil or Lamivudine/Tenofovir disoproxil, NRTI) on four interleukins (IL-4, IL-10, IL-13, and IL-21) as immune activation markers in naïve HIV(Human Immunodeficiency Virus)-infected patients during the first six months of combined standard-of-care antiretroviral therapy (cART). Newly diagnosed with HIV-infected subjects and without any disease that could affect the immune activation markers were evaluated. The patients’ physicians recommended the cART as standard-of-care and the ILs were measured before cART and six months of cART. The levels of CD4^+^ T-cells count and CD4^+^/CD8^+^ ratio significantly increased at six months (P-value<0.02) regardless of the drugs, INSTIs or PI. However, a CD4^+^/CD8^+^ >1 was observed in 25% of patients treated with Raltegravir and half of those treated with Dolutegravir. At six months of cART, viral load was detectable in only 6/31 individuals. IL-21 had an undetectable level in 30/31 patients after six months of cART. Our results suggest the potency in restoring immune markers in HIV-infected patients with all investigated drugs. Dolutegravir showed a tendency to statistically significant changes in IL-4 and IL-10. A clinical trial with random allocation of medication and an extensive follow-up is needed to replicate this research and validate the usefulness of evaluated ILs as markers of cART effectiveness.

## Introduction

Infection with human immunodeficiency virus (HIV) is an important public health problem all over the world. The diagnosis of HIV infection is delayed due to long-time subclinical manifestations, and thus the infected persons can spread the virus unwittingly. This issue affects public health policies and represents the main barrier to HIV pandemic control. According to World Health Organization (WHO), almost thirty-eight million persons are estimated to live in 2020 with HIV infection [1]. The number of newly infected patients slightly reduced to 1.5 million, and the number of deceased persons due to HIV-related causes reduced to 680,000 in 2020 [1]. Introducing combined antiretroviral therapy (cART) was the most crucial advance in treating this infection, leading to the transformation into a chronic disease. The cART suppresses the virus’s multiplication, maintains a good state of health for the patients, and proves highly effective in reducing the HIV transmission [2]. Thus, cART constitutes an essential strategy in transmitting, preventing, and controlling HIV-associated morbidity and mortality.

WHO’s recommends the initiation of cART as soon as possible after the diagnosis [3, 4]. The treatment can reduce the viral load to an undetectable level while partially restoring the immunological function. Under cART, HIV-associated mortality is decreased while non-HIV-associated diseases, such as stroke, cardiovascular and metabolic diseases, are increased [5, 6].

The pathogenesis of HIV infection is not entirely understood. The virus affects the cells with CD4 receptors on the surface, like T-helper lymphocytes, macrophages, mononuclear cells, impairing the immune system’s proper function. The impairment in the function of CD8^+^ cells is maintained by residual HIV replication and gut bacterial translocation [7], which enables a persistent antigen load [8–10] even with a suppressed viral load induced by cART. Thus, a continuous expression of inhibitory receptors like PD-1 (programmed death receptor-1), TIM-3 (T cell immunoglobulin and mucin domain-containing protein-3), LAG-3 (lymphocyte activation Gene-3), CD160, 2B4 (CD244) [11], and the secretion of inflammatory mediators [12–14] is maintained.

A chronic inflammatory response characteristic to HIV infection is seen in the absence of an efficient viral response and elimination of the virus. This chronic inflammation is associated with functional changes of immune cells [15], a decrease in antiviral response [16], failure of immune reconstruction under cART [17], and organ damage [18]. The immune activation mechanism in HIV infection is explained by persistent viral replication, loss of the gut mucosa’s integrity, and increased proinflammatory cytokines. The immune activation level can predict an accurate CD4^+^ T-cell depletion than viral load [19] and the risk of developing AIDS-associated co-morbidities and mortality [20, 21]. IL-4 promotes the differentiation of CD4^+^ T-cells into the Th2 phenotype, a process also mediated by IL-10 and IL-13 [22]. IL-10 had increased levels in acute HIV infection [23], while IL-21 correlated with relative control of HIV replication [24]. IL-6, a proinflammatory cytokine, had plasma levels depending on individual variability, non-HIV related factors (e.g., age, smoking and co-morbidities [25, 26]), and thus, the plasma concentration cannot be associated mainly with HIV infection. The C-reactive protein (CRP), a marker widely studied due to the frequency of determination at the point of care, showed higher values in HIV-infected persons along with D-dimer and IL-6 [26, 27].

Limited information on the effect of different cARTs on interleukins levels is available in the scientific literature and mainly refers to HIV-infected persons under cART [28, 29]. It has been demonstrated that cART initiation decreases systemic inflammatory markers regardless of the class, but rarely achieved levels are comparable to HIV-uninfected individuals [30]. The life expectancy increased after starting cART at age 20, with an addition of 43.3 years in high-income countries and 22.9 years in low/middle-income countries [31]. The effectiveness of raltegravir (RAL), atazanavir/ritonavir (ATV/r), or darunavir/ritonavir (DRV/r) associated with TDF/FTC (tenofovir disoproxil fumarate emtricitabine) on viral suppression has been demonstrated in naïve-HIV infected patients [32]. No consistent evidence regarding the reduction of inflammation (e.g., hsCRP (high-sensitivity C-reactive protein), IL-6, and D-dimer) between different ART regimes were reported [32].

Our pilot observational study aimed to investigate the effects of standard-of-care cART on four markers of immune activation, namely IL-4, IL-10, IL-13, and IL-21 evaluated before and at six- months of cART.

## Materials and methods

The study was conducted according to the guidelines of the Declaration of Helsinki and approved by The Ethical Committee of the Iuliu Hațieganu University of Medicine and Pharmacy, Cluj-Napoca (approval no. 144 from 02/04/2018).

### Study design and data sources

A cohort study with prospective data collection was conducted at the Cluj-Napoca AIDS Center, Romania, from June 2018 to January 2020 on patients newly diagnosed with HIV infection. Newly diagnosed patients with HIV infection, without previous cART medication (cART-naïve) and absence of any other viral or bacterial infection were eligible for the study. Patients newly diagnosed with HIV infection but with cardiovascular disease, diabetes, malignancies, other systemic inflammatory diseases, or AIDS-defining conditions found at baseline evaluation were excluded. The eligible patients were invited to participate and those who agreed were included in the study after they signed informed consent.

The Cluj-Napoca AIDS Center has patients mainly from the five counties of the North-West region of Romania, namely, Cluj, Bihor, Maramureş, Satu Mare, and Sălaj, under medical care since 1990. Since 2014, this center’s addressability has increased and patients from different Romanian counties (such as Hunedoara, Sibiu, Alba, and Bistriţa-Năsăud) are also registered. The cART is initiated after specific evaluation, generally within one week after diagnosis, thus leading to patients’ higher treatment adherence, better control of disease progression, and reduction in community transmission of HIV infection.

Demographic data (age, gender), mode of infection (heterosexual, men who have sex with men—MSM or parenteral such as intravenous drug users—IDU), and history of other viral infections (hepatitis B, hepatitis C, cytomegalovirus) were collected.

The patients were diagnosed according to the criteria of the Centers for Disease Control and Prevention 1993 (revised in 2008) surveillance case definition [33] and to the guideline of the European Center for Disease Control [34]. The HIV infection was classified in three clinical stages (A, B, and C) and three immunological stages (stage 1: CD4^+^ count >500/mm^3^, stage 2: CD4^+^ count is between 200/mm^3^ and 500/mm^3^, and stage 3: CD4^+^ <200/mm^3^).

According to the European AIDS Clinical Society [35], the treatment regimens are elaborated by the patient’s physicians and include two Nucleoside Reverse-Transcriptase inhibitors (NRTIs: Emtricitabine/Tenofovir disoproxil or Lamivudine/Tenofovir disoproxil) and a third agent, either an INSTI (Dolutegravir, Raltegravir or Elvitegravir) or a boosted PI (Darunavir/r). Naïve HIV-infected patients had a psychological evaluation before cART initiation as standard-of-care. The boosted PI is prescribed to patients with high grade of anxiety or symptoms of depression that does not need medication. The INSTI is prescribed to all other patients, according to the availability of the drugs in the hospital pharmacy at the moment of cART initiation and the patient’s personal preference after discussing the possible adverse effects of each INSTI drug. The cART regimens are the standard-of-care and are chosen by the patients’ physicians, according to drug availability in the hospitals’ pharmacy at the beginning of the treatment and the compliance of the patient (the potential to induce depression or lead to suicidal ideation, weight gain, increased serum lipid values, frequency of administration and number of tablets/dose/day, the need of an association with a meal). All patients started their treatment within one week after the diagnosis.

### Sample collection and measurements

C-Reactive Protein (CRP), HIV viral load, CD4^+^ and CD8^+^ count, interleukin-4 (IL-4), interleukin-10 (IL-10), interleukin-13 (IL-13), and interleukin-21 (IL-21) were measured at the time of diagnosis and after six months of cART for all patients included in the study.

Blood was drawn by vein puncture from all subjects who agreed to participate in the study. The blood was collected with k3-EDTA for CD4^+^, CD8^+^ T cells, and viral load. HIV viral load was evaluated using polymerase chain reaction—(COBAS AmpliPrep/COBAS TaqMan HIV-1 v2.0 test, Roche, Switzerland) with an analytical sensitivity of 40 copies/mL and 100% specificity.

Interleukins 4 (IL-4), 10 (IL-10), 13 (IL-13), and 21 (IL-21) were determined by ELISA with IBL kit (R&D Systems, USA). Four milliliters of venous blood were collected into serum separator tubes, centrifugated for 30 minutes, and then stored at -20°C. The used dilution was 1:2. The limits of detection were as follows: 1.3 pg/mL for IL-4, 1 pg/mL for IL-10, 0.7 pg/mL for IL-13, and 20 pg/mL for IL-21.

### Statistical analysis

The evaluated markers were reported as median (Q1 to Q3) (where Q is the quartile) since the number of subjects per subgroup is small. We reported the median {minimum to maximum} whenever the number of measurements was less than 5. Differences between baseline and six months follow-up were tested with the Wilcoxon matched-pairs test for the whole cohort. In support of the paired analysis, data were reported for all available participants and for paired subjects. Mann-Whitney test was used to compare independent groups. The Kruskal Wallis test was used to compare markers’ baseline levels on different classes of drugs. Spearman’s rank correlation coefficient was used for the association analysis between CD4^+^ T-cell levels, CD4^+^/CD8^+^ and viral load on the one hand, and levels of interleukins on the other. The measurements lower than the detection limit were reported as undetectable data to avoid data fabrication and bias. Statistical package (v. 13.5, StatSoft, OK, USA) was used to analyze data. A P-value smaller than 0.05 was considered statistically significant, considering a significance level of 5% in the context of an exploratory analysis.

## Results

### Clinical and demographic characteristics

From June 2018 to July 2019, in our center, a total of 95 patients were diagnosed with HIV infection, 77 of them being male, and 46 of them were MSM and/or bisexual. Unfortunately, only 31 patients fulfilled the eligibility criteria, were enrolled from June 2018 to July 2019, and thus followed up no later than January 2020.

All eligible population members represented by thirty-one subjects, aged at diagnosis from 19 to 54 years, were included in the study. With one exception, the study participants were male, the most frequent way of HIV transmission was MSM, and most subjects were in stage A of disease (Table 1).

**Table 1:** Main characteristics of the subjects (n = 31).

Characteristic	Value
Median age, (Q1 to Q3) years	27 (23.5 to 32)
Male sex, no. (%)	30 (96.8)
Urban living, no. (%)	20 (64.5)
County, no. (%)	
Cluj-Napoca	13 (41.9)
Others	18 (58.1)
HIV transmission, no. (%)	
MSM	19 (61.29)
Heterosexual	11 (35.48)
IDU	1 (3.23)
Stage of HIV infection, no. (%)	
A1	8 (25.8)
A2	15 (48.4)
A3	1 (3.2)
B1	3 (9.7)
B2	4 (12.9)

Q1 = first quartile; Q3 = third quartile. MSM = men who have sex with men. IDU = intravenous drug user; no. = absolute frequency; % = percentage; A, B—clinical stages of HIV infection; stage 1 (CD4^+^ count>500/mm^3^, stage 2 (CD4^+^ count 200-500/mm^3^), stage 3 (CD4^+^ count<200/mm^3^)–immunological stage of HIV infection according to WHO classification.

### Markers’ levels: Baseline vs. six months of cART

The investigated patients showed a significant increase in CD4^+^ and CD4^+^/CD8^+^ ratio values after treatment, thus indicating the overall treatment’s effectiveness in downregulating CD4^+^ destruction and diminishing CD8^+^ recruitment in HIV chronic infection (Table 2). Proinflammatory IL-21 was detectable at follow-up in one subject under treatment with Darunavir/r, who had a viral load also still detectable (292.79 IU/mL).

**Table 2:** Levels of evaluated markers among HIV-infected subjects before cART and 6 months of cART.

Evaluation Marker	Baseline	6 months of cART	Stat. (*p*-value)
**CD4**^**+**^ **(/mm**^**3**^**)**	416 (310 to 545.5), 31	633 (490.5 to 796.5), 31	4.5 (<0.0001)
**CD4** ^**+**^ **/CD8** ^**+**^	0.42 (0.28 to 0.63), 31	0.74 (0.56 to 1.12), 31	4.8 (0.0192)
**Interleukins**			
IL-4 (pg/mL)	4.74 (3.74 to 7.27), 31		
	4.74 (3.69 to 7.35), 30	4.00 (3.65 to 8), 30	0.01 (0.9914)
IL-10 (pg/mL)	6.73 (3.43 to 8.69), 31		
	5.65 (3.30 to 9.00), 27	4.06 (2.78 to 7.37), 27	0.10 (0.9234)
IL-13 (pg/mL)	3.36 (2.99 to 5.33), 10	1.23 (1.00 to 2.18), 8	
	2.94 (2.33 to 5.71), 5	1.81 (1.03 to 3.29), 5	0.13 (0.8927)
IL-21 (pg/mL)	807 (435.81 to 118.28), 15		
	787.55, 1	517.36, 1	n/a

Values are expressed as median (Q1 to Q3), n; where Q1 is the 25^th^ percentile and Q3 is the 75^th^ percentile, n is the number of patients; Stat. stands for the te statistics of the Wilcoxon matched-pairs test. n/a = not available.

The C-reactive protein levels at diagnosis had normal values in all individuals (lower than 1 mg/dL, median = 0.15, (Q1 to Q3) = (0.07 to 0.20)). Viral load before cART varied from 887 IU/ml to 9846512 IU/mL (median = 156049, (Q1 to Q3) = (23535.5 to 607223.5)). Most individuals had a baseline viral load <100.000 IU/mL (15/31), followed by those with values >500.000 IU/mL (9/31).

Significant monotonic associations had been identified only between CD4^+^ T-cells, CD4^+^/CD8^+^ ratio, viral load (IU/mL), and IL-10 (Table 3).

**Table 3:** Significant or tendency to statistical significance: Correlations between CD4^+^ T cells, CD4^+^/CD8^+^ ratio, CRP, viral load (IU/mL), and ILs in HIV-infected subjects before the commencement of cART.

Markers	Spearman’s ρ (P-value), n
**CD4**^**+**^ **(/mm**^**3**^**) vs. IL-10 (pg/mL)**	-0.38 (0.0350), 31
**CD4**^**+**^**/CD8**^**+**^ **vs. CRP (mg/dL)**	-0.47 (0.0081), 30
**CD4**^**+**^**/CD8**^**+**^ **vs. IL-10 (pg/mL)**	-0.61 (0.0003), 31
**Baseline viral load (IU/mL) vs. IL-10 (pg/mL)**	0.39 (0.0279), 31

#### Integrase strand transfer inhibitors vs. boosted protease inhibitor

In most of the cases, the applied third cART was an INSTI (22/31, either Raltegravir 15/31, or Dolutegravir 4/31, or Elvitegravir 3/31). A boosted PI (boosted Darunavir) was administrated in 9/31 subjects. No differences were observed when baseline viral load on INSTIs groups was compared to PI group (Table 4). Only three patients in the INSTIs group and three in the PI group had a detectable viral load at follow-up evaluation (Table 4) without significant differences in cases with follow-up detectable viral load between groups (Fisher exact test, P-value = 0.2326). The CD4^+^/CD8^+^ significantly increased while the values of IL-4 decreased (Table 4) for all patients treated either with an INSTI or PI. The CD4^+^ significantly increased in the INSTI group, while the PI group achieved only a tendency to statistical significance (Table 4).

**Table 4:** Levels of evaluated markers amongst HIV-infected subjects before cART and 6 months of cART(follow-up) in INSTIs vs. PI.

	Evaluation	INSTIs	PI	Stat. (P-value)
**Age, years**	Baseline	24 (22.3 to 29.5), 22	31 (27 to 45), 9	1.8 (0.0709) ^b^
**CD4**^**+**^ **/mm**^**3**^	Baseline	428 (305 to 547.3), 22	394 (339 to 436), 9	-0.02 (0.9826)^b^
	Follow-up	696.5 (503.8 to 802.8), 22	590 (457 to 669), 9	-0.98 (0.3275)^b^
	Stat (P-value)^a^	3.9 (0.00008)	1.9 (0.0506)	
**CD4** ^**+**^ **/CD8** ^**+**^	Baseline	0.48 (0.30 to 0.70), 22	0.41 (0.27 to 0.43), 9	-0.67 (0.5)^b^
	Follow-up	0.94 (0.57 to 1.22), 22	0.63 (0.56 to 0.70), 9	-1.41 (0.1573)^b^
	Stat (P-value)^a^	4.1 (0.00005)	2.4 (0.0152)	
**Viral load (IU/mL)**	Baseline	275497 (33443 to 675901), 22	70099 (18849 to 191278), 9	1.20 (0.2314)
	Follow-up	550 (387 to 1606), 3	293 (199 to 328), 3	n/a
	Stat (P-value)	n/a	n/a	
**IL-4 pg/mL**	Baseline	5 (4 to 6.9), 22	4.4 (3.7 to 10.9), 9	-0.37 (0.7114)^b^
	Follow-up	4 (3.7 to 9.2), 19	4.2 (3.8 to 5.5), 9	0.38 (0.7005) ^b^
	Stat (P-value)^a^	0.04 (0.9702)	0.06 (0.9528)	
**IL-10 pg/mL**	Baseline	6.7 (3.4 to 9.4), 22	7.2 (3.7 to 7.9), 9	0.3 (0.7773)
	Follow-up	5.6 (3.3 to 10.9), 19	6.4 (3.1 to 8), 8	
		4.2 (3.3 to 10), 19	3.2 (1.1 to 4.3), 8	-1.4 (0.1757)
	Stat (P-value)^c^	0.2 (0.8092)	0.8 (0.408)	
**IL-13 pg/mL**	Baseline	13/22	8/9	n/a (0.2310) ^c^
	Follow-up	16/22	7/9	n/a (0.8488) ^c^
	Stat (P-value)^a^	n/a (0.4531)	n/a	
**IL-21 pg/mL**	Baseline	14/22	2/9	n/a (0.0873) ^c^
	Follow-up	22/22	8/9	n/a (0.5806) ^c^
	Stat (P-value)^c^	n/a	n/a	

Data are reported as median (Q1 to Q3), where Q is the quartile excepting IL-13 and IL-21 where the number of cases with undetectable levels is reported.

^a^ Wilcoxon matched pairs test

^b^ Mann-Whitney test

^c^ Fisher’s exact test; n/a = not available; Stat = statistics of the test.

**Fig 1:**
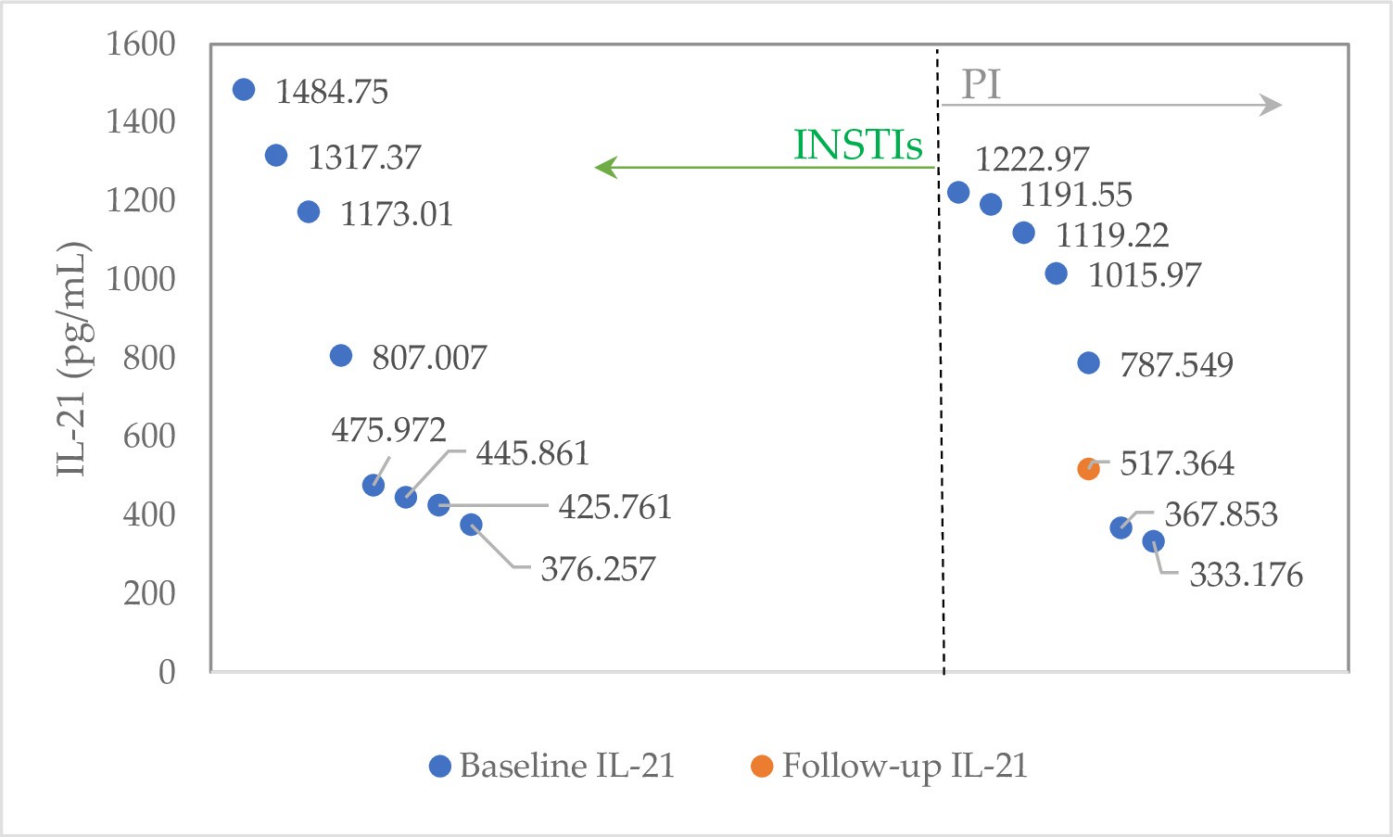
The change in IL-21 levels baseline vs. follow-up grouped by the third cART.

IL-21 was detectable in 8 subjects with INSTIs and 7 subjects with PI at baseline and remained detectable only in one patient who received PI (Fig 1).

#### Raltegravir vs. dolutegravir vs. elvitegravir vs. boosted darunavir

The comparisons of subjects with different third drug (Raltegravir, Dolutegravir, Elvitegravir, or boosted Darunavir) showed no statistically significant differences neither at baseline nor at six months follow up in regards of CD4^+^ (/mm^3^) (P-values>0.5), CD4^+^/CD8^+^ (P-values>0.1), viral load (IU/mL) (P-value = 0.4481 baseline), IL-4 (pg/mL) (P-values>0.1), IL-10 (pg/mL) (P-values>0.10), IL-13 (pg/mL) (P-values>0.2), or IL-21 (pg/mL) (P-value = 0.8831, baseline). Regardless of the third cART, IL-21 becomes undetectable in almost all patients (Table 5).

**Table 5:** Levels of evaluated markers amongst HIV-infected subjects before cART and 6 months of cART by the used drug.

	Baseline	6 months follow-up	Stat. (P-value)
***Raltegravir (n = 15)***			
CD4^+^ (/mm^3^)	364 (250.5 to 507.5), 15	668 (490.5 to 773.5), 15	-3.3 (0.0010)
CD4^+^/CD8^+^	0.35 (0.25 to 0.55), 15	0.93 (0.58 to 1.14), 15	3.4 (0.0007)
IL-4 (pg/mL)	5.7 (4.4 to 7.3), 15		
	5.7 (4.5 to 7.4), 14	4.6 (3.7 to 9.6), 14	0.8 (0.4216)
IL-10 (pg/mL)	8.7 (4.2 to 13.3), 15		
	8.2 (3.5 to 15.7), 12	6.8 (4.1 to 37.4), 12	1.2 (0.2393)
IL-13 (pg/mL)	3.2 (3 to 3.9), 6		
	3.2 {1.0 to 25.7}, 3	1.6 {1 to 89}, 3	n/a
IL-21 (pg/mL)	896.7 {376.3 to 1484.8}, 4	15/15 undetectable	n/a
***boosted Darunavir (n = 9)***			
CD4^+^ (/mm^3^)	394 (339 to 436), 9	590 (457 to 669), 9	2.0 (0.0506)
CD4^+^/CD8^+^	0.41 (0.27 to 0.43), 9	0.63 (0.56 to 0.74), 9	2.4 (0.0152)
IL-4 (pg/mL)	4.4 (3.7 to 10.9), 9	4.2 (3.8 to 5.5), 9	0.06 (0.9528)
IL-10 (pg/mL)	7.2 (3.7 to 7.9), 9		
	6.4 (3.1 to 8.0), 8	3.2 (1.1 to 4.3), 8	0.8 (0.4008)
IL-13 (pg/mL)	n/a, 1	n/a, 2	n/a
IL-21 (pg/mL)	1016 (577.7 to 1155.4), 7	8/9 undetectable	n/a
***Dolutegravir (n = 4)***			
CD4^+^ (/mm^3^)	635 {440 to 696, 4	819.5 {781 to 861}, 4	1.8 (0.0679)
CD4^+^/CD8^+^	0.8 {0.56 to 0.93}, 4	1.29 {0.67 to 1.45}, 4	1.8 (0.0679)
IL-4 (pg/mL)	4.8 {4.4 to 7.5}, 4	3.7 {2.9 to 3.7}, 4	1.8 (0.0679)
IL-10 (pg/mL)	5.3 {2.6 to 6.6}, 4	3.4 {2.1 to 4.1}, 4	1.8 (0.0679)
IL-13 (pg/mL)	4.6 {3.5 to 5.7}, 2	0.8 {0.6 to 1}, 2	n/a
IL-21 (pg/mL)	445.9 {425.8 to 807}, 3	4/4 undetectable	n/a
***Elvitegravir (n = 3)***			
CD4^+^ (/mm^3^)	416 {301 to 509}, 3	521 {498 to 594}, 3	n/a
CD4^+^/CD8^+^	0.5 {0.22 to 0.52}, 3	0.48 {0.41 to 0.97}, 3	n/a
IL-4 (pg/mL)	3.7 {3.7 to 3.7}, 3	4 {3.5 to 10}, 3	n/a
IL-10 (pg/mL)	3.3 {1.2 to 6.9}, 3	0.8 {0.4 to 7.3}, 3	n/a
IL-13 (pg/mL)	8.7 {8.7 to 8.7}, 1	3/3 undetectable	n/a
IL-21 (pg/mL)	2/3 undetectable	3/3 undetectable	n/a

Data are expressed as median (Q1 to Q3), n or median {minimum to maximum}, n–where Q is the quartile and n is the number of subjects. Stat is the statistics of the Wilcoxon matched pairs test was used for comparison. n/a = not available.

The levels of all investigated ILs have decreased regardless of the used drug, with a tendency to significance only in patients treated with Dolutegravir. A significant increase of CD4^+^ levels was observed in patients with Raltegravir, while those who received boosted Darunavir or Dolutegravir showed a tendency to statistical significance (Table 5). Twenty-five percentage of subjects who received Raltegravir showed a follow-up CD4^+^/CD8^+^ higher than one.

The HIV viral load was not detectable in 13/15 patients who received Raltegravir, 6/9 patients who received boosted Darunavir, 3/4 patients who received Dolutegravir, and all patients (3/3) who received Elvitegravir.

## Discussion

The results of our study suggest a significant increase in CD4^+^ count and CD4^+^/CD8^+^ ratio at six months of cART, with CD4^+^/CD8^+^ ratio higher than one in 25% of patients that received Raltegravir or Dolutegravir. The ILs levels decrease regardless of the third cART drug, showing potential restoration of the immune system’s HIV-associated inflammation.

Our sample included mainly men (Table 1) because at the moment of enrollment, from a total of 18 women diagnosed over this period, only 7 of them were in clinical stage A or B, most of them being in an advanced stage of HIV infection (AIDS) with additional active diseases and thus not eligible for this study. The advanced stage of HIV infection in women has been previously reported on our population [36]. Consequently, in our study, almost all our patients were men, and the primary way of transmission was MSM, which is not similar to data reported for the Romanian population with a predominance of heterosexual transmission [37]. The difference between our study and our country can be explained by the fact that our region is under continuous socio-cultural, scientific, and industrial development, with a tendency of behaviorism towards western European countries [38].

The INSTIs regimen is the first-line third cART drug due to their antiviral efficacy, good tolerability, and few drug-drug interactions [39]. Although antiretroviral treatment may reduce the levels of inflammation markers [40], most of them remain increased for years in people living with HIV infection, despite viral replication suppression and reconstruction of CD4^+^ cells [41]. In our study, the CD4^+^ cell count has increased in half of the patients with 217/mm^3^ and the CD4^+^/CD8^+^ ratio increased from 0.42 to 0.74, indicating that the immune reconstruction process was under restoration even after only six months of cART (Table 2). Our results are similar to reported data from a multicentre cohort study [42]. The immune restoration was seen in all treated groups at six months of cART, increasing CD4^+^ cells and CD4^+^/CD8^+^ ratio (Tables 4 and 5). The Dolutegravir group had higher baseline CD4^+^ levels and higher CD4^+^ levels at six months (Table 5), suggesting minor immune dysfunction at baseline. The effects on CD4^+^ and CD4^+^/CD8^+^ were similar regardless of the third cART drug (Tables 4 and 5), increasing CD4^+^ and CD4^+^/CD8^+^, and decreasing of all investigated ILs, suggesting a tendency towards no immune imbalance in naïve HIV-infected patients (Table 5).

Most patients obtained an undetectable viral load at six months of cART (25/ 31 patients, see Table 4). The observed high rate in the treatment’s response could be explained by the patient’s acceptance of the disease and recommended cART, the prompt response of the physicians in resolving any associated medical issues, and the constant psychological support.

We found differences in serum levels of inflammatory biomarkers between patients treated with INSTIs or PI (Table 4). We found slight differences in IL’s serum levels between different INSTI regimens (Table 5), but these differences did not reach statistical significance. IL-4 has previously been shown to stimulate HIV expression in promonocytic cell lines and primary human monocytes by upregulating the expression of HIV mRNA [43]. Increased IL-4 levels were also correlated with a high replication rate in HIV-infected children [44]. Similar to prior reported results in which IL-4 gradually decreased after cART initiation [45], we have also found a decrease of IL-4 after Raltegravir, Dolutegravir, and boosted Darunavir (Table 5).

IL-10, a prototypical anti-inflammatory cytokine, plays a key role in HIV-associated immune dysregulation by having an important immunosuppressive effect. Its serum levels are positively associated with increased viral load, and disease progression and are decreased by effective antiretroviral therapy [46–48]. In our study, IL-10 serum levels have been significantly correlated with CD4^+^ count, CD4^+^/CD8^+^ ratio, and viral load at baseline (Table 3). Among INSTIs, Dolutegravir decreased the IL-10 levels with a tendency toward statistical significance (Table 5), but this must be carefully interpreted since it reflects only four patients. A decrease of IL-10 values at six months and one year of cART had also been reported by Ouiros-Roldan et al. [49], but was statistically significant only in patients with INSTIs.

IL-13 and IL-4 have similar anti-inflammatory effects in modulating monocyte function but have divergent effects on HIV expression in monocytes. Mikovits et al. [50] reported that IL-4 initially increases HIV viral expression, then stimulates cytolysis of HIV-infected monocytes, while IL-13 initially decreased acute HIV viral infection, but in the long term, could no longer suppress HIV expression in monocytes. Our pilot study found that IL-13 levels were undetectable for 2/4 of patients treated with Dolutegravir and all patients treated with Elvitegravir (Table 5).

IL-21 has pleiotropic effects, modulating mature lymphocytes’ function, thus playing an essential role in controlling chronic HIV viral infection. Early in the infection course, cytokine production is compromised, serum levels in infected patients correlate with CD4^+^ T cell count. Furthermore, circulating HIV-specific IL-21-producing CD4^+^ T-cells are lower in HIV-infected individuals than in healthy controls [51]. Iannello et al. reported on HIV-infected patients an association of CD4^+^ T-cell with IL-21 and between clinical stage and HAART (Highly Active Antiretroviral Therapy–today’s cART) [52]. Iannello et al. also reported an IL-21 increase progressively after antiviral treatment initiation with HAART [52]. Similar results have also been reported by Zheng et al. [53]. No recent study evaluating IL-21 levels in HIV-infected individuals before and after cART commencement has been published according to our availability to the scientific publications. We have found that IL-21 had undetectable levels in 30/31 treated patients (Table 5). The only patient with persistent IL-21 levels was treated with boosted Darunavir, who also had a detectable viral load at six months follow-up. This finding could be explained by the fact that some patients’ viral load drops slower under treatment.

HIV-infected patients display imbalanced immunological alterations of different cytokine serum levels. Combined ART has proven to increase the CD4^+^ cells, improve the CD4^+^/CD8^+^ and decrease the viral load, a context for longer and healthier life. Even though the life expectancy has improved, cART does not entirely restore health, maybe due to insufficient knowledge regarding the impact on different cytokines and their evolution pattern under treatment. Our results suggest the beneficial effects of different third cART on serum levels of IL-4, -10, -13, and -21.

This pilot study was not a clinical trial but used a convenience sample. However, all eligible population agreed to participate and was evaluated, minimizing the selection bias. The sample was limited by the study’s design and we made substantial efforts to include only patients without any other diseases that could affect the value of evaluated ILs. Furthermore, the COVID-19 pandemic restricted access to the healthcare of HIV-infected subjects, and thus we limited the follow-up evaluation to six months of cART. A clinical trial with random allocation of medication, standardized doses, existance of a control group and extensive follow-up (e.g., one year, two years, five years) is needed to replicate this research and validate the usefulness of evaluated ILs as markers of cART effectiveness.

The reported results reflect what was observed on the evaluated sample, and the results’ generalizability is not appropriate. However, our results encourage the study’s extension considering a larger multicenter sample with random cART allocation. Despite the heterogeneity of the baseline IL-21 levels between groups (Table 5), undetectable values are observed at six months follow-up, so maybe a more sensitive laboratory measurement method could appropriately reveal the effects of cART on this IL. Measurement of ILs could be helpful in current medical practice once their evolution in time is validated as predictors for treatment effectiveness, clinical events and/or HIV-associated co-morbidities. Consistent evidence in reducing inflammation and immune activation is thus required for evaluating the impact of different cART regimens.

## Conclusions

Our study suggests a potency in restoring immune markers in HIV-infected patients with all investigated drugs. CD4^+^ T-cells and CD4^+^/CD8^+^ ratio significantly increased at follow-up evaluation regardless of the third cART but with a tendency to statistical significance for IL-4 and IL-10 on patients who received Dolutegravir. IL-21 was the only studied IL that had decreased in all patients, showing undetectable levels in most cases after six months of cART, and may be a investigated as a predictor of treatment effectiveness. However, more research is needed to validate the results of this pilot study. First, a multicenter clinical trial is needed with random allocation of cART and balanced numbers of subjects per regimes. Second, the control participants would be of great value in the context of ILs evaluation. Third, an extensive follow-up evaluation to prove the long-term effect of cART on immune restoration could lead to identifying the best performing regime.
